# Considerations of Privacy and Confidentiality in Developing a Clinical Support Tool for Adolescent Tobacco Prevention: Qualitative Study

**DOI:** 10.2196/12406

**Published:** 2019-04-28

**Authors:** Ryan P Theis, Ali M Malik, Lindsay A Thompson, Elizabeth A Shenkman, Lori Pbert, Ramzi G Salloum

**Affiliations:** 1 Institute for Child Health Policy Department of Health Outcomes and Biomedical Informatics University of Florida Gainesville, FL United States; 2 College of Medicine University of Florida Gainesville, FL United States; 3 Department of Pediatrics University of Florida Gainesville, FL United States; 4 Department of Population and Quantitative Health Sciences University of Massachusetts Medical School Worcester, MA United States

**Keywords:** clinical decision support, adolescent, primary care, tobacco use, confidentiality, implementation science, qualitative research

## Abstract

**Background:**

Electronic clinical support tools show promise for facilitating tobacco screening and counseling in adolescent well-care. However, the application of support tools in pediatric settings has not been thoroughly studied. Successfully implementing support tools in local settings requires an understanding of barriers and facilitators from the perspective of both patients and providers.

**Objective:**

This paper aimed to present the findings of a qualitative study conducted to inform the development and implementation of a support tool for adolescent tobacco screening and counseling in 3 pediatric clinics in North Florida. The primary objective of the study was to test and collect information needed to refine a tablet-based support tool with input from patients and providers in the study clinics.

**Methods:**

A tablet prototype was designed to collect information from adolescents on tobacco susceptibility and use before their well-care visit and to present tobacco prevention videos based on their responses. Information collected from adolescents by the support tool would be available to providers during the visit to facilitate and streamline tobacco use assessment and counseling components of well-care. Focus groups with providers and staff from 3 pediatric clinics (n=24) identified barriers and facilitators to implementation of the support tool. In-depth interviews with racially and ethnically diverse adolescent patients who screened as susceptible to tobacco use (n=16) focused on acceptability and usability of the tool. All focus groups and interviews were audio-recorded and transcribed for team-based coding using thematic analysis.

**Results:**

Privacy and confidentiality of information was a salient theme. Both groups expressed concerns that the tool’s audio and visual components would impede privacy and that parents may read their child’s responses or exert control over the process. Nearly all adolescents stated they would be comfortable with the option to complete the tool at home via a Web portal. Most adolescents stated they would feel comfortable discussing tobacco with their doctor. Adolescent interviews elicited 3 emergent themes that added context to perspectives on confidentiality and had practical implications for implementation: (1) *purity*: an expressed lack of concern for confidentiality among adolescents with no reported history of tobacco use; (2) *steadfast honesty*: a commitment to being honest with parents and providers about tobacco use, regardless of the situation; and (3) *indifference*: a perceived lack of relevance of confidentiality, based on the premise that others will “find out anyway” if adolescents are using tobacco.

**Conclusions:**

This study informed several modifications to the intervention to address confidentiality and introduce efficiency to well-care visits. The support tool was integrated into the electronic health record system used by the study clinics and modified to offer videos to all adolescents regardless of their tobacco use or susceptibility. Future studies will further test the acceptability of the intervention in practice.

## Introduction

### Background

Primary care providers (PCPs) play an important role in screening, counseling, and early intervention for adolescent tobacco use [1]. Brief interventions by PCPs, as recommended by the United States Preventive Services Task Force (USPSTF), can reduce the risk of tobacco initiation in adolescents [2,3]. However, PCP practices for tobacco screening and counseling are inconsistent, with studies reporting low rates of physician adherence to evidence-based practices for smoking cessation and for routine screening of adolescent electronic cigarette (e-cigarette) use [4,5]. Barriers to effective counseling by PCPs can include limited time and lack of privacy during the visit, whereas possible facilitators include checklists for adolescents to complete before the visit and assurances of confidentiality by the PCP [6,7].

Electronic clinical support tools show promise for promoting adolescent tobacco screening and counseling, in part because they help clinics overcome barriers and leverage facilitators to well-care. Such tools have become increasingly common in behavioral health interventions as a means of facilitating patient-provider communication and ensuring thorough and consistent application of evidence-based practices [8]. Although there is evidence for the effectiveness of support tools for adolescent substance abuse screening in primary care [9], the more general application of support tools to the adolescent population remains to be thoroughly explored and documented. Furthermore, the successful implementation of support tools can depend on their capacity to integrate into clinical workflow, appropriateness to patient populations, competing clinical priorities, and other local contextual factors [10].

### Objectives

To address the challenges that PCPs face with regard to limited time in well-care visits and concerns about privacy and confidentiality, we developed an electronic tool to support PCPs in adolescent tobacco screening and counseling. The tool includes a survey about tobacco use, susceptibility, and concerns that adolescents can complete before their well-care visit, educational videos tailored to the responses they provide, and electronic transmission of their responses to their PCP to facilitate counseling. These design elements can help overcome gaps in the consistent application of evidence-based practices for adolescent tobacco screening and counseling, which can in turn lead to reductions in tobacco use initiation and promote tobacco cessation.

This paper has presented the findings from a qualitative pilot study conducted to refine the design and content of the support tool and understand the context needed to effectively implement it in local clinical settings. In particular, we sought to elicit the perspectives of PCPs and adolescent patients on the confidentiality of information that adolescents provide about their tobacco use in well-care visits. Triangulating the responses of both types of end users, this study informed important modifications to the support tool to address concerns about privacy and improve the support tool’s acceptability and feasibility for implementation.

## Methods

### Setting

This study was conducted in collaboration with pediatric primary care clinics in the University of Florida (UF) Health System, which serve urban and rural communities in North Florida and represent a diverse patient population with regard to income, education, and race/ethnicity [11]. The UF Health System is a member of the OneFlorida Clinical Research Consortium—a research collaborative that includes a centralized cooperative institutional review board, shared governance, and implementation support from a network of community practice facilitators and local providers [12].

### Intervention

**Figure 1:**
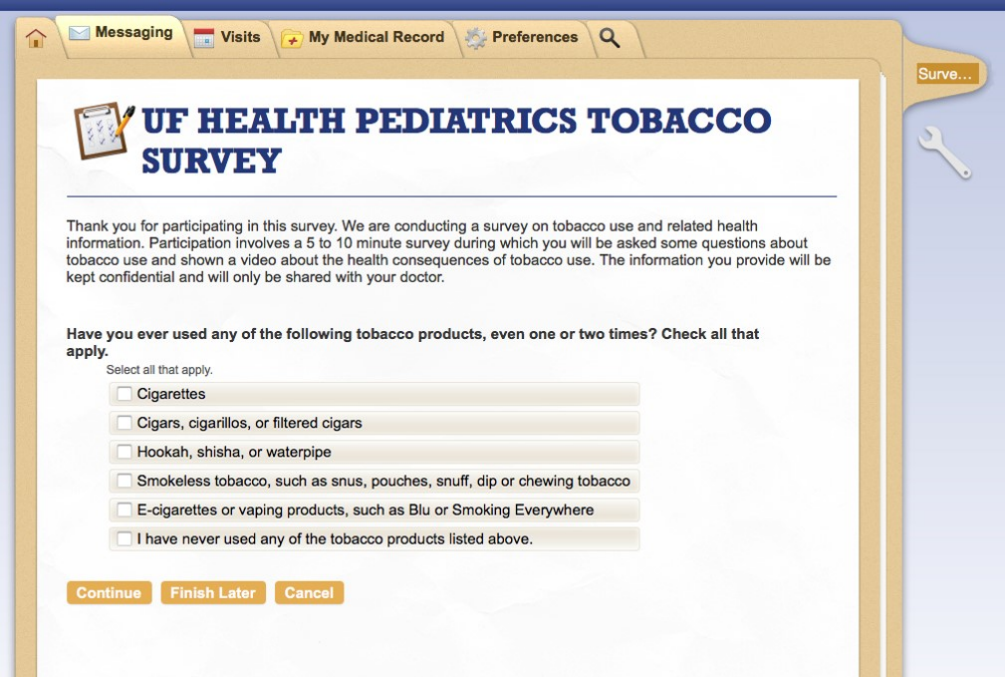
Screenshot of the electronic clinical support tool: tobacco use screener.

Following a stakeholder engagement approach that included methodologists, clinicians, and community representatives, we developed an initial prototype of an electronic tobacco screening tool, which includes a questionnaire for adolescents to complete before their scheduled well-care visit (see prototype slides in Multimedia Appendix 1). The questionnaire begins with an initial screening to assess the adolescent’s history of tobacco use, collecting separate information on 5 types of nicotine and tobacco product classes (cigarettes, cigars/cigarillos, hookah, smokeless tobacco, and e-cigarettes), as shown in Figure 1. For nonusers, the tool assesses susceptibility to any tobacco products, indicated by a lack of firm commitment to avoid tobacco use [13]. Specifically, the susceptibility screener asks whether the adolescent would use a cigarette, e-cigarette, or other tobacco product (1) if offered by a friend, (2) at any time in the next 12 months, and (3) 5 years from the time of screening.

**Figure 2:**
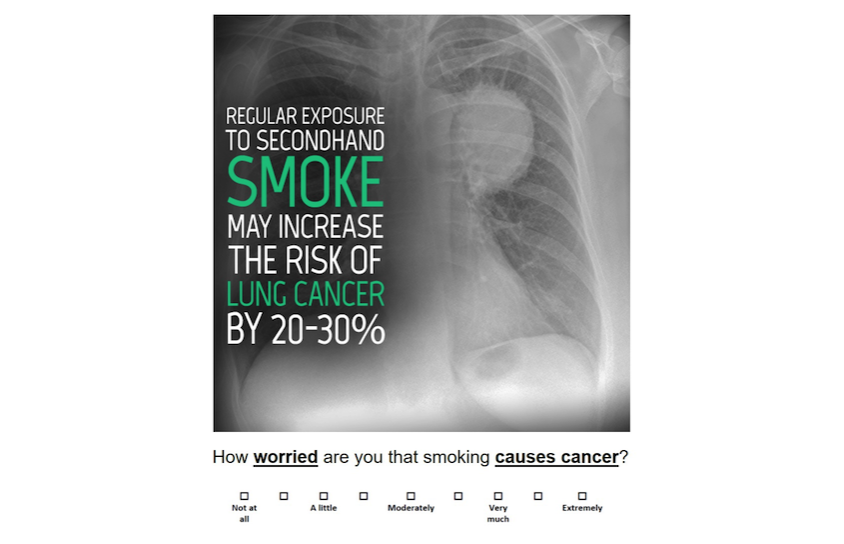
Screenshot of the electronic clinical support tool: tobacco consequences rating.

Although the questions in the screening tool are similar to what may be asked in a standard preclinical visit survey, their collection in electronic format allows for additional functions to facilitate tobacco screening and counseling—including options to complete the tool using a Web-based portal (outside the clinical setting), the presentation of educational content, and transmission of responses to PCPs. For both users and susceptible nonusers, the tool presents a list of consequences of using tobacco products and asks them to rate which consequences they are most likely to ask their doctor about (see example in Figure 2).

Then, based on their pattern of responses, the tool presents 1 brief educational video from the US Food and Drug Administration tobacco prevention campaign, which can supplement and optimize face-to-face counseling provided by the PCP [14]. For adolescents with a history of tobacco use, the video applies to the tobacco product that the adolescent selected; in the case of multiple selected products, the selection algorithm prioritizes videos based on relative harm (eg, combustible products taking precedence over e-cigarettes). For nonusers who screen as susceptible to tobacco, the video is based on their selection of tobacco use consequences. The decision to limit the tool to a single video was based on the need to keep the screening brief and minimize user burden. Finally, questionnaire responses are made available to providers to prime them to further counsel adolescents on tobacco use.

### Data Collection

The support tool was further tested through collaboration with pediatric clinics in the UF Health system. Our qualitative approach included (1) focus groups with providers and staff to identify barriers and facilitators to implementation and (2) in-person qualitative interviews with adolescent patients to test the usability of the tool. The focus groups and interviews were conducted sequentially between July 2016 and June 2017 to obtain provider and patient perspectives on a working prototype of the tool in iterative stages.

The provider focus groups were conducted in 3 different clinics, with topics selected based on Proctor’s Framework for Implementation Outcomes—focusing specifically on acceptability, appropriateness, adoption, and feasibility of the tool within the context of clinical workflow [15]. All focus groups included a presentation of the tool to participants and were facilitated by the study’s qualitative investigator. Focus groups lasted 45 min on average and were audio-recorded for later transcription and analysis.

In-depth interviews were conducted with adolescents (aged 12 to 17 years) recruited from the UF Adolescent Clinic, which serves racially and ethnically diverse patients with a high rate of Medicaid coverage (80%) [11]. Adolescents meeting the age criteria who had a visit to the clinic during the study period were eligible for inclusion and invited to participate by the study’s clinician investigator. All assenting patients were later contacted by telephone and administered a brief tobacco use history and susceptibility screener (the same screeners used in the support tool, as shown in Multimedia Appendix 1). Those who indicated a history of tobacco use or nonusers who screened as susceptible to tobacco use were scheduled for in-person interviews. Adolescents with no history of tobacco use and who did not screen as susceptible were not eligible for the study; this exclusion was made because many important features of the tool we sought to test—including the tobacco use consequence rating and the educational video—are completed only by adolescents who report using or being susceptible to tobacco.

Interview topics included (1) adolescents’ perceptions and attitudes about tobacco products, (2) their personal experiences using or being offered tobacco products, and (3) their opinions of the prototype support tool. Discussions about the tool focused on the ease of use, comprehension of content, and assessment of acceptability and appropriateness to the target population. Adolescents were asked to discuss how comfortable they would be talking with their doctor about using tobacco, completing the screening in the presence of their parent (eg, in the clinic’s waiting room), and completing the screening in a more private setting (eg, using a Web-based patient portal). Interviews were led by 2 interviewers trained in qualitative data collection methods and were audio-recorded for later transcription and analysis. Parents provided informed consent for their child’s participation but were not physically present during the interviews. The research protocol was approved by the UF Institutional Review Board.

### Data Analysis

For both focus group and interview transcripts, analyses involved an initial phase of deductive coding (using categories derived from the moderator guides), a secondary phase of inductive coding (to identify emerging themes), and an interpretive phase in which findings from both data types were synthesized [16]. This approach allowed for the iterative development of separate codebooks for the provider focus groups and adolescent interviews, whereby transcripts were back-coded with any new themes identified during the inductive phase. To ensure high interrater reliability, each transcript was coded independently by 2 trained coders and then reviewed in team meetings to discuss and obtain consensus on coding discrepancies [17]. Team meetings included, at minimum, both coders and a third study investigator who was familiar with the content but had not participated in coding. Independently coded transcripts were reviewed side by side, and all coding discrepancies were discussed and resolved by consensus. In the event that the coders could not come to a consensus on a particular coding discrepancy, the third team member made the final determination. The analysis was conducted using NVivo 11 (QSR International, 2015).

In addition, a framework analysis method was used to organize the findings from the adolescent interviews [18]. A summary template was developed for initial review of transcripts, in which content could be abstracted for domains specific to the study aims (eg, support tool acceptability, message relevance, and comfort discussing with doctor). Completed summaries were reviewed in regular team meetings and compiled into a descriptive participant-by-domain matrix [19]. As a complementary analytic strategy, the team reviewed the final matrix to assess patterns in domain responses across interview participants.

To facilitate the final, interpretive phase of the analysis, queries for codes related to support tool acceptability and appropriateness (which applied to both study populations) were produced for the provider and adolescent transcripts separately. These queries were reviewed for emergent themes relevant to the 2 types of implementation outcomes, and commonalities and contrasts in themes were documented between providers and adolescents.

## Results

### Provider Perspectives

The focus groups were each attended by 7 to 9 participants (24 participants overall), including physicians, midlevel providers (physician assistants and nurse practitioners), and office staff (Table 1).

Confidentiality of patient information was a salient theme in all focus groups. In early phases of the study, the waiting room was the setting proposed for adolescents to complete the support tool (in tablet format) to minimize disruption of clinical workflow. Providers in the first 2 focus groups voiced concerns with this approach, calling upon their past experiences with patient intake forms. As 1 provider remarked:

Most of the time we give out our handout, it’s supposed to be filled out by the patient. But the parent takes it and they fill it out for them. So how do we prevent that from happening?

In cases where adolescents might complete the screening on their own, providers still had concerns about confidentiality, noting that parents would be able to see the tablet screen (including their child’s responses to the questionnaire) and hear the videos. They expected that adolescents would not respond honestly to the tobacco susceptibility and history questions. This issue raised questions about the feasibility of the intervention:

We do a physical survey that 99% of the time, the parents are watching them do. So, I am not sure how effective it is, and I am not sure how honest they are… Then, you have a video that now confirms that they probably said that they smoked and that the parent can now hear, even if the teenager can somehow hide it from them. I would worry about that as a barrier.

As a possible solution, providers proposed separating the adolescent from the parent before administering the screener. However, this came with its own concerns, as some clinics may not have a separate room or area where adolescents could complete the tool privately, posing an important barrier to broader implementation. More relevant to the question of confidentiality, some providers also noted that parents would question why their child was being brought aside and what the staff were asking them:

We have parents who respect that, and we have parents who absolutely get very angry if we start talking to their teenager about keeping things from them.

Another proposed solution was to integrate the tool into a Web-based patient portal that was at the time being expanded to adolescent populations in the UF Health System pediatric clinics. The portal would allow adolescents to complete the screening on their own computer or mobile device at home before their visit. For adolescent patients aged 12 years and older who have enrolled in the portal, only they would have access to their information on the portal, allowing for the privacy necessary to ensure confidentiality.

**Table 1:** Provider focus group participants.

Focus group participants	Clinic number 1	Clinic number 2	Clinic number 3
	July 2016	April 2017	June 2017
Physician (n)	3	3	3
Registered nurse/Licensed practical nurse (n)	4	2	3
Advanced practice registered nurse/Physician assistant (n)	0	1	2
Office staff (n)	0	2	1

On the basis of this feedback, the support tool was modified to be used through the portal —allowing adolescents the option to complete the tool at home or in the office using the original tablet intervention. In both cases, the tool was integrated with the electronic health record (EHR) system used by the clinics, allowing providers real-time access to the information provided by the adolescents. These modifications were tested in the third provider focus group, who generally perceived them as positive. One provider remarked on several potential advantages of the intervention, including the promotion of clinical efficiencies:

I think that adolescents are going to feel… like their privacy is better protected if they can [do it] on their iPhone, their iPad, or at home prior to the clinic visit… It’s an ultimate timesaver. It’s not adding something if it’s more efficient. It has to be done only once. It auto-populates our charts and can be done beforehand when a child’s at home and potentially in a more private situation. We might get more accurate information as well.

Nevertheless, providers in the third focus group maintained concerns related to parents’ access to information. Although the portal is confidential for adolescents, parents may still “bully” their children to gain access and they would be more likely to see the responses on a larger screen. With regard to the waiting room intervention, providers stated that some parents would offer to complete the screening themselves, on behalf of their children—echoing concerns expressed in the earlier focus groups.

Finally, providers in all focus groups noted that certain behavioral characteristics of adolescents could impede the feasibility of the intervention, regardless of the setting or mode of administration. One provider commented on adolescents’ reluctance to self-report weaknesses or to talk about risky behaviors—raising the question of whether the more private Web portal mode would actually encourage more honest responses. Other providers suggested that some adolescents would become bored with the tool, as evident in the following exchange:

After four or five screens, they’re probably going to check out at that point. They’re just going to be clicking.

Provider 1

Then they’re just going to start Christmas Treeing, trying to get through it.

Provider 2

### Adolescent Perspectives

Among 128 adolescent patients who initially agreed to participate, 65 could not be reached after 3 attempts (51%), 46 were ineligible to participate because they were nonsmokers who did not screen as susceptible to tobacco (36%), 1 refused to participate (1%), and 16 participated in an interview (13%).

Participants represented a fairly even mix of female and male patients (56% and 44%, respectively) and included patients of black (38%), Hispanic (25%), white (19%), and other (19%) racial and ethnic groups (Table 2). All participants initially screened as being susceptible to tobacco or nicotine products; however, only 2 (13%) reported any active tobacco use during the interview.

**Table 2:** Adolescent interview participant characteristics.

Characteristics		Statistics, n (%)
**Age (years)**		
	12	1 (6)
	13	4 (25)
	14	2 (13)
	15	3 (19)
	16	4 (25)
	17	2 (13)
**Gender**		
	Female	9 (56)
	Male	7 (44)
**Race and ethnicity**		
	Black, non-Hispanic	6 (38)
	Hispanic	4 (25)
	Other, non-Hispanic	3 (19)
	White, non-Hispanic	3 (19)
**Ever used tobacco or nicotine product**		
	No	14 (88)
	Yes	2 (13)
**Ever been offered tobacco or nicotine product**		
	No	10 (63)
	Yes	6 (38)
**Comfortable discussing tobacco with doctor**		
	No	0 (0)
	Yes	9 (56)
	Maybe	1 (6)
	Unknown^a^	6 (38)
**Comfortable using support tool near parents**		
	No	4 (25)
	Yes	12 (75)
**Would complete support tool honestly in a home setting**		
	No	1 (6)
	Yes	15 (94)

^a^Six adolescents were not asked whether they would feel comfortable talking with their doctor about tobacco after using the support tool.

#### Using the Tool in Clinical Settings

Nearly all adolescents stated they would be less comfortable using the tool in the clinic’s waiting room than in a more private setting. Moreover, 2 participants specifically expressed concerns with the tool’s media components if they were to use it in the waiting room. Both remarked that the sounds and images of the video would be noticed by others, which might discourage them from responding honestly. However, 1 adolescent acknowledged that once in the examination room, the tool could help adolescents who are uncomfortable with face-to-face interactions. She noted that, by asking many of the questions about tobacco use that a doctor might otherwise ask, the tool could help ease and streamline the visit:

That’ll get them away from talking face-to-face with the problem they might be having, and then the doctor or your counselor going straight to the questions they need to be asking.

Participant 5, aged 14 years, female, black

Most adolescents stated that they would feel comfortable discussing tobacco with their doctor. Several mentioned they were already comfortable with their doctors, suggesting that a level of rapport necessary for honest discussions had been built during the course of previous visits with the same provider. Others stated they would disclose tobacco use with their doctor because they expected confidentiality or because they considered disclosure to be in their best interest. When asked why he would be comfortable talking about tobacco with his doctor, 1 adolescent replied:

Because I will probably most likely learn something new. And it would give me a better chance to understand why people like to do it.

Participant 1, aged 17 years, male, Hispanic

#### Using the Tool at Home

Nearly all adolescents stated a preference for using the tool via the Web portal at home. Some indicated that completing the screening at home would afford them more privacy and encourage them to respond more honestly. When asked her opinion of using the tool at home, 1 adolescent responded positively, stating:

Because I feel like at a doctor’s office or somewhere else you’re always like, “Oh, who’s watching me?” And at home you have the privacy of your own.

Participant 6, aged 17 years, female, Hispanic

However, 1 adolescent stood out from the others in acknowledging that the in-home portal option could lead to a less honest response. A private setting is also an unsupervised setting, with little to encourage adolescents to actually engage with the tool, read its content, and provide thoughtful responses.

I wouldn’t think that I would answer them more honestly at home, but rather click and skim through it… They don’t have people asking, really, for the truth, and they can just be scrolling through it and clicking whatever they think is right.

Participant 1, aged 17 years, male, Hispanic

#### Nuances of Disclosure—Purity, Steadfast Honesty, and Indifference

In many cases, adolescents did not agree that the Web-based option would encourage them to respond more honestly—primarily because they would already have responded honestly in any format or setting. Overall, 3 distinct themes emerged that helped to explain this finding. First, a theme of purity was observed among the majority of adolescent participants who were never smokers, many of whom stated they had “nothing to hide.” Anticipating no potentially sensitive responses to the tool, these adolescents reported they would be equally comfortable completing it in the waiting room or at home. The presence of parents was not a barrier because parents “already know” how their children would respond to the questions about personal use. Several adolescents acknowledged that although they would have no hesitations in using the tool, other peers who used tobacco might have hesitations. As 1 adolescent stated:

I know I wouldn’t answer any differently, but friends of mine probably would, and other people my age just ‘cos they have more to hide about it. I don’t really care much ‘cos I’m not smoking... Their parents – if they see anything that they’ve answered, they could get in trouble and just the presence of their parents being there would be intimidating for them.

Participant 3, aged 16 years, female, white

Second, many participants commented on the value of steadfast honesty—a commitment to provide honest responses regardless of the situation. Steadfast honesty was connected to positive relationships with parents and with perceptions of well-being. As 1 adolescent stated:

I feel like nobody should be afraid to talk about things… It’s better to talk about things to people instead of holding it in.

Participant 14, aged 16 years, female, other

Third, several adolescents expressed a sentiment of indifference toward privacy and confidentiality of sensitive information. Some stated that they would respond honestly because they were not concerned with who might read their responses. Others remarked that their parents were “nosy” and would find out their responses anyway. As 1 adolescent remarked:

If I smoke tobacco, I would tell them because, I mean, they’re my parents. Eventually, they’re gonna find out. Well, it doesn’t matter if I did it or didn’t, either way, they will know.

Participant 2, aged 16 years, male, Hispanic

## Discussion

### Principal Findings and Comparison With Previous Work

Both providers and adolescents in this study stressed the importance of privacy and confidentiality for successful implementation of the clinical tool. Confidentiality was considered essential for encouraging adolescents to use the screener and to ensure honest responses among those who completed it. Themes relevant to the parental influence also emerged, including the control exerted by parents over the screening process. These findings are consistent with a similar qualitative study that found confidentiality and parental influence to be important in adolescent perspectives of comprehensive risk assessments [20].

When used in a waiting room in tablet format, the tool has features that can easily be noticed by others, and the presence of parents may lead adolescents to underreport their tobacco use and risk. Consequently, the majority of adolescents expressed a preference for the Web-based version and reported that they would respond to the screener more honestly at home. This contrasts with findings of a study by Jasik et al [21], in which adolescents preferred completing behavior screening using a tablet in the waiting room, rather than at home. It is likely that adolescents’ preference for the Web-based tool in our study is related to the more intrusive audio and video components of the intervention. A recent study on the same confidential adolescent portal used in our study (MyChart) also found that adolescents consistently used the portal after enrollment [22]. Although based on the response of a single adolescent, there remains the possibility that unsupervised use of the Web-based tool at home may not result in complete or honest responses, which is a concern that warrants further study.

Some providers also expressed concerns that parents might complete the screener for their child or question the child’s need for privacy. Although the prospect of a Web-based version alleviated some of these concerns, some providers maintained that parents might “bully” their children to gain access to the portal. However, the extent to which these actions by parents may constitute the norm is unclear. A study by Miller et al [23] of parental perspectives on factors influencing adolescent communication with physicians found that most parents valued their adolescent having time alone with their physician.

As the majority of adolescent participants were never-smokers, it is unsurprising that they would express few concerns about confidentiality of information about their smoking history. However, these participants screened as tobacco-susceptible for the study, likely making them a higher-risk group. This suggests that adolescents may perceive less risk in disclosing their intent to use tobacco, supporting the inclusion of susceptibility questions in screening tools. Assessment of susceptibility is important and should be utilized in future screening tools, given that greater than one-quarter of never-smoking adolescents are susceptible for future tobacco use as adults and rates of susceptibility to tobacco products increase with age [24].

The theme of steadfast honesty clarifies adolescent perceptions of disclosure in clinical settings, as many expressed a commitment to provide honest responses regardless of the setting or situation. Most stated they would feel comfortable discussing tobacco with their doctor—a finding that is consistent with the USPSTF recommendation for PCP-led tobacco interventions [2]. This study revealed pathways that may encourage adolescents to discuss tobacco with their doctors, including having a positive patient-provider relationship, an understanding of the value of disclosure for health promotion, and a curiosity about the physiological effects of tobacco and the psychosocial factors behind its use. Regardless of their tobacco use status, wellness visits may represent a learning opportunity for adolescents that can help them make healthier choices. Furthermore, it is possible that the tool may itself increase adolescents’ trust in their doctors and foster more positive relationships—an association that has been found in similar interventions with adult populations and that requires further study among adolescents [25].

Several modifications to the intervention were made from our iterative approach. First, the tool is now integrated into the EHR system used by the study clinics, introducing efficiency to the well-care visit and adding value to the learning health system. The tool is bundled with the American Academy of Pediatrics Bright Futures health risk assessment—an important adaptation that streamlines the intervention with existing clinical practice. Second, the tool offers videos to all adolescents regardless of their tobacco use or susceptibility. Patients and their parents learn that the videos are offered to all clinic patients as part of its preventive services, thereby mitigating concerns about confidentiality.

### Strengths and Limitations

This study has several strengths and limitations. The perspectives of multiple stakeholders were considered throughout the intervention’s design, enhancing its utility. Furthermore, triangulation of sources with both patients and providers increased credibility of the qualitative findings, permitting a comprehensive understanding of acceptability and perceived feasibility of the intervention by key users [26]. These data were collected before implementation, which allowed stakeholder feedback to increase the intervention’s chances of success. However, this also introduced a limitation in that concerns by participants had not yet been tested or validated in practice. Future phases will study the acceptability of the intervention after implementation, permitting a more focused evaluation of the degree to which confidentiality may be a concern.

The study included purposeful samples of participants, allowing for in-depth understanding of the perspectives of stakeholders in clinical settings where the intervention is targeted. Although the generalizability of findings to other populations is not an aim of qualitative work, it is important to acknowledge how findings may or may not be transferable to similar, specific contexts [26,27]. The decision to integrate the tool with the adolescent’s EHR was appropriate for this study’s clinics, which are part of a large academic health center. In clinical settings that are not part of a similar collaborative, providers may be less willing to support such integration out of concerns for protecting confidentiality of sensitive information *within* the EHR [28].

The study’s focus on adolescents who screened as susceptible was a strength. However, our findings were limited by the small number of adolescents who reported any history of tobacco use—an important subgroup whose perspectives may not have been sufficiently explored. Furthermore, because this information was based on self-reporting, the extent to which smoking history may have been underreported is unknown. Thematic saturation was reached within the 16 adolescents overall, with no new themes emerging after review of the fifth transcript. However, it is unlikely that saturation was reached specifically for adolescents who reported a history of tobacco use. This subgroup will be more fully included in future phases of this study.

### Conclusions

In summary, we found commonalities and differences between provider and patient perspectives on the confidentiality of information collected in an electronic clinical support tool for adolescent tobacco screening and counseling. The resulting intervention allows PCPs to have expedient access to reliable information on susceptibility and tobacco use history during adolescent well-care visits. The intervention can both enhance counseling for active tobacco users and provide content to potentially prevent tobacco uptake among adolescents who screen as susceptible. Future studies are planned to further test the acceptability of the intervention in practice and will include adolescents across the full spectrum of tobacco use and susceptibility.

## Appendix

Multimedia Appendix 1 Tobacco counseling support tool prototype used in focus groups and interviews.

## Abbreviations

e-cigarette: electronic cigarette
EHR: electronic health record
PCP: primary care provider
UF: University of Florida
USPSTF: United States Preventive Services Task Force
