# Supplementary material for: Considerations of Privacy and Confidentiality in Developing a Clinical Support Tool for Adolescent Tobacco Prevention: Qualitative Study
Source: JMIR Form Res. 2019 Apr 28;3(2):e12406. doi: 10.2196/12406 (PMC6528437; doi:10.2196/12406)
Supplement: Multimedia Appendix 1 [file formative_v3i2e12406_app1.pptx]

## Slide 1
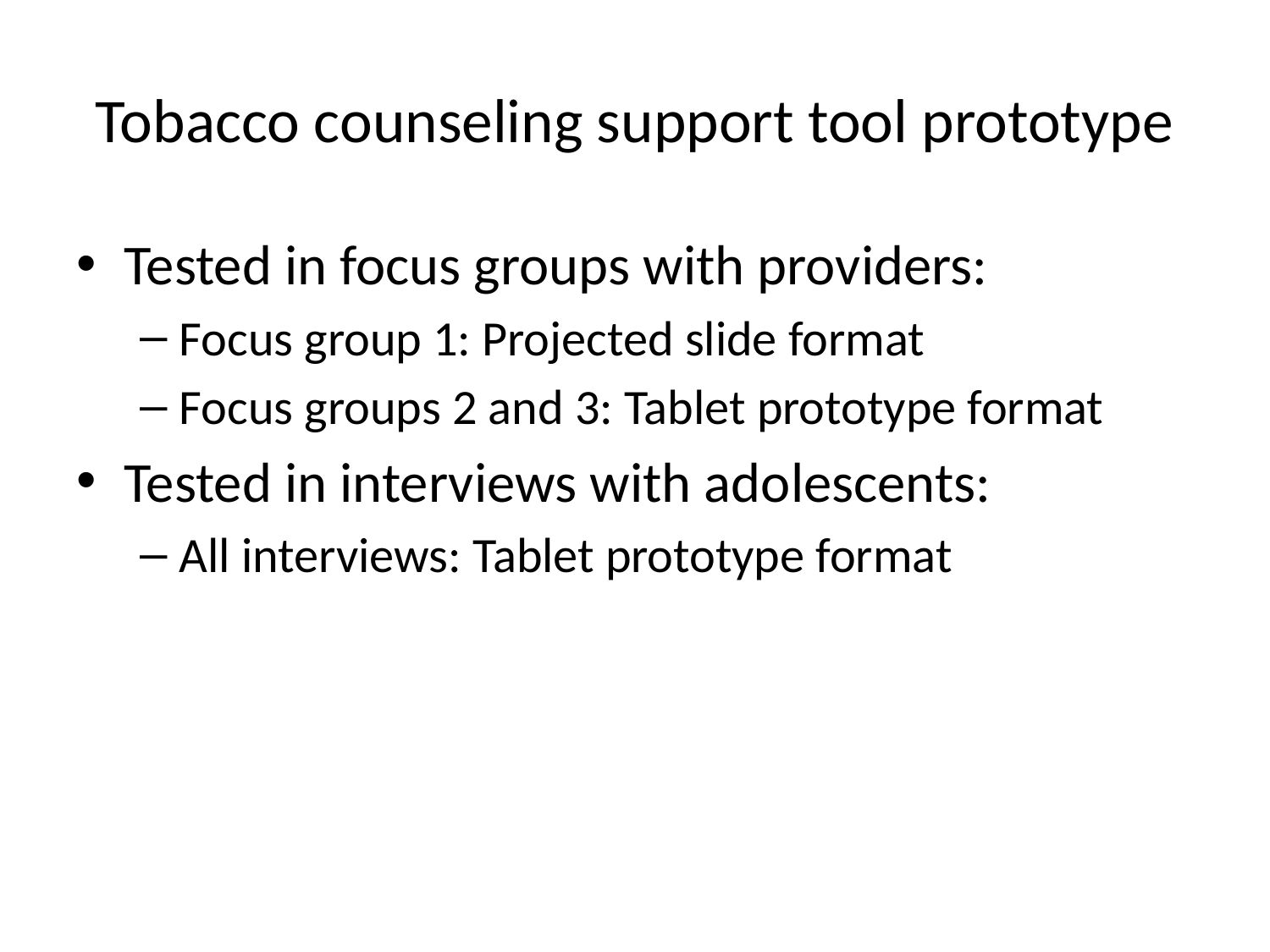

# Tobacco counseling support tool prototype
Tested in focus groups with providers:
Focus group 1: Projected slide format
Focus groups 2 and 3: Tablet prototype format
Tested in interviews with adolescents:
All interviews: Tablet prototype format

## Slide 2
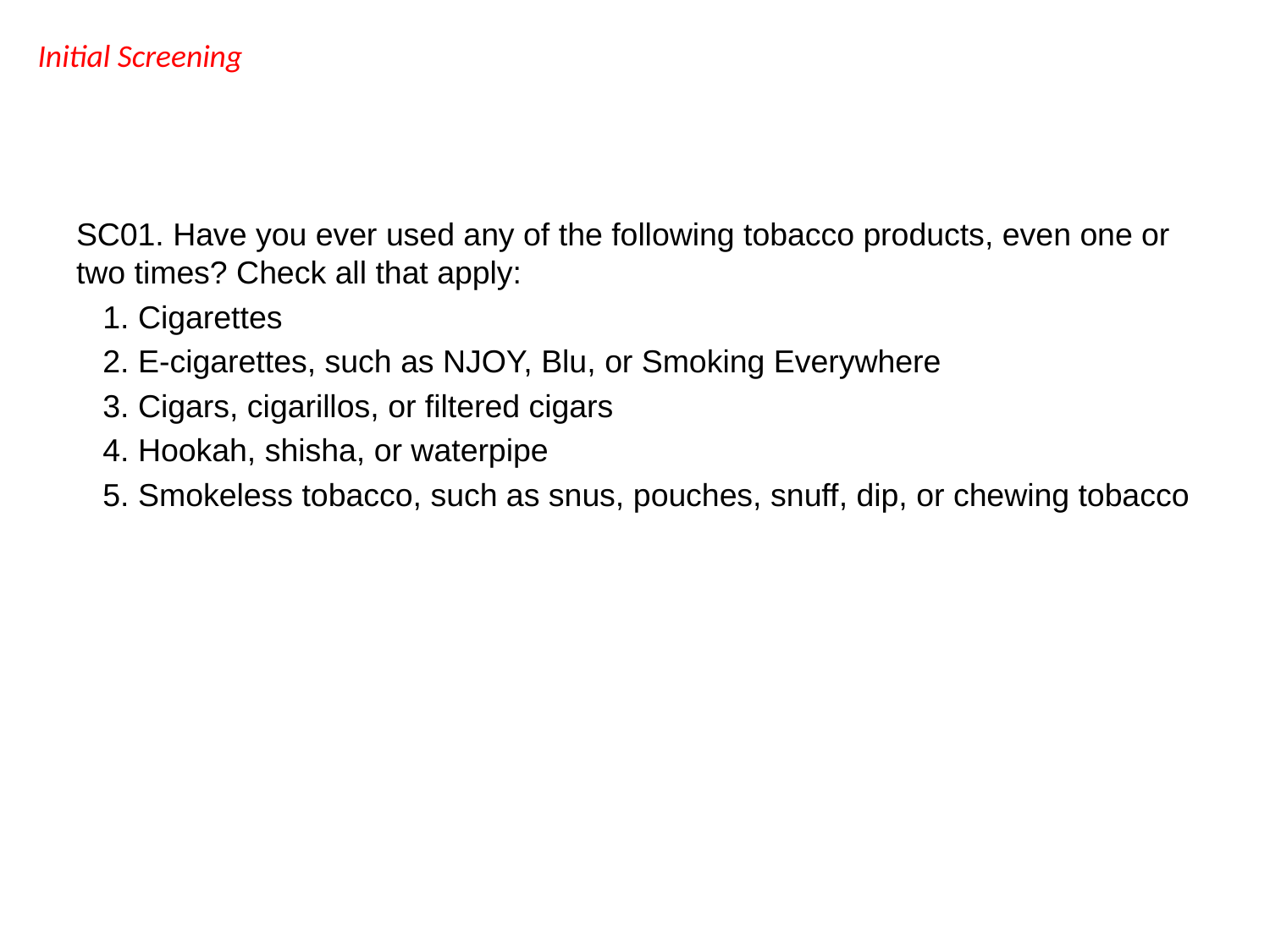

Initial Screening
SC01. Have you ever used any of the following tobacco products, even one or two times? Check all that apply:
 1. Cigarettes
 2. E-cigarettes, such as NJOY, Blu, or Smoking Everywhere
 3. Cigars, cigarillos, or filtered cigars
 4. Hookah, shisha, or waterpipe
 5. Smokeless tobacco, such as snus, pouches, snuff, dip, or chewing tobacco

## Slide 3
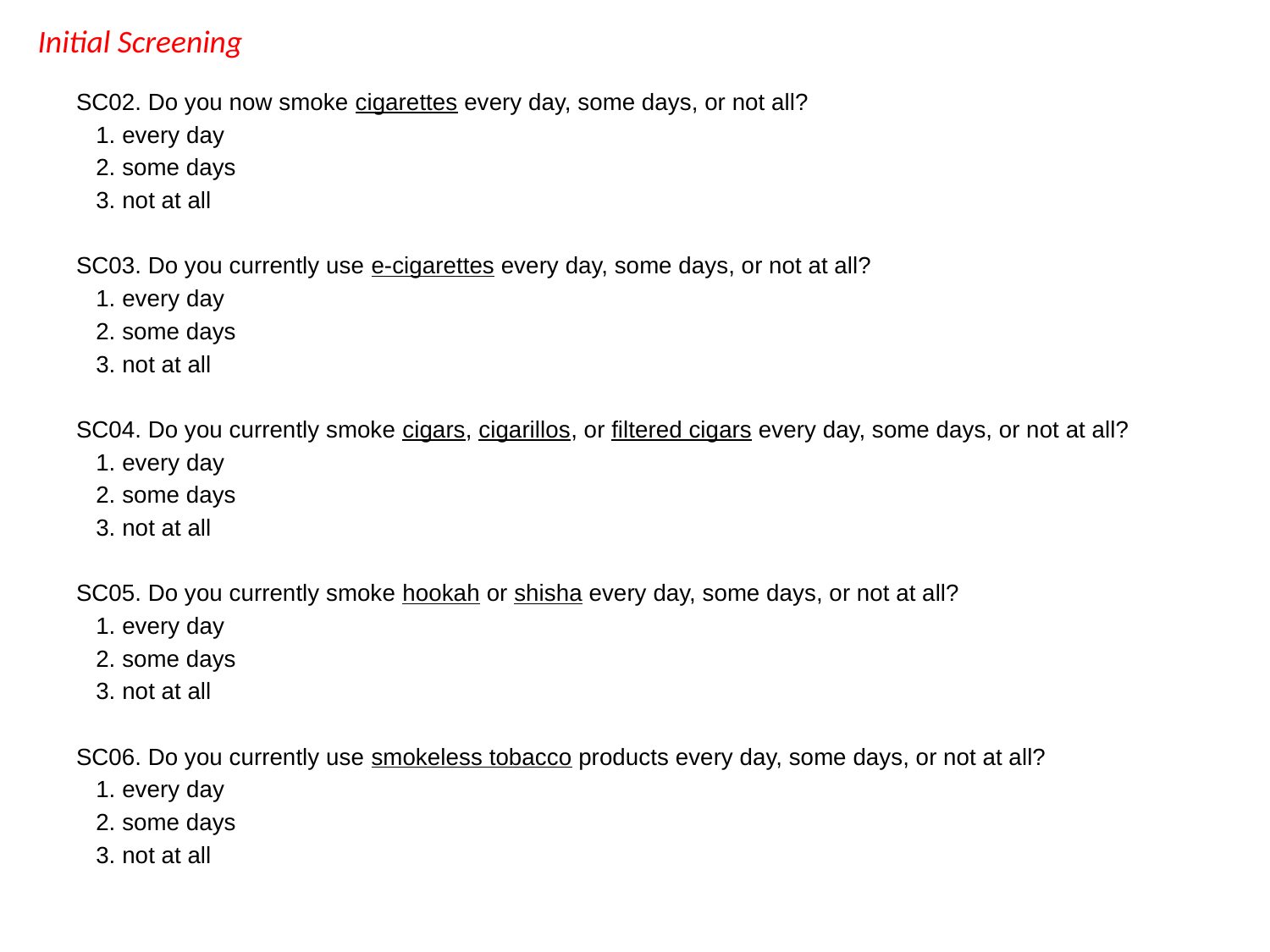

Initial Screening
SC02. Do you now smoke cigarettes every day, some days, or not all?
 1. every day
 2. some days
 3. not at all
SC03. Do you currently use e-cigarettes every day, some days, or not at all?
 1. every day
 2. some days
 3. not at all
SC04. Do you currently smoke cigars, cigarillos, or filtered cigars every day, some days, or not at all?
 1. every day
 2. some days
 3. not at all
SC05. Do you currently smoke hookah or shisha every day, some days, or not at all?
 1. every day
 2. some days
 3. not at all
SC06. Do you currently use smokeless tobacco products every day, some days, or not at all?
 1. every day
 2. some days
 3. not at all

## Slide 4
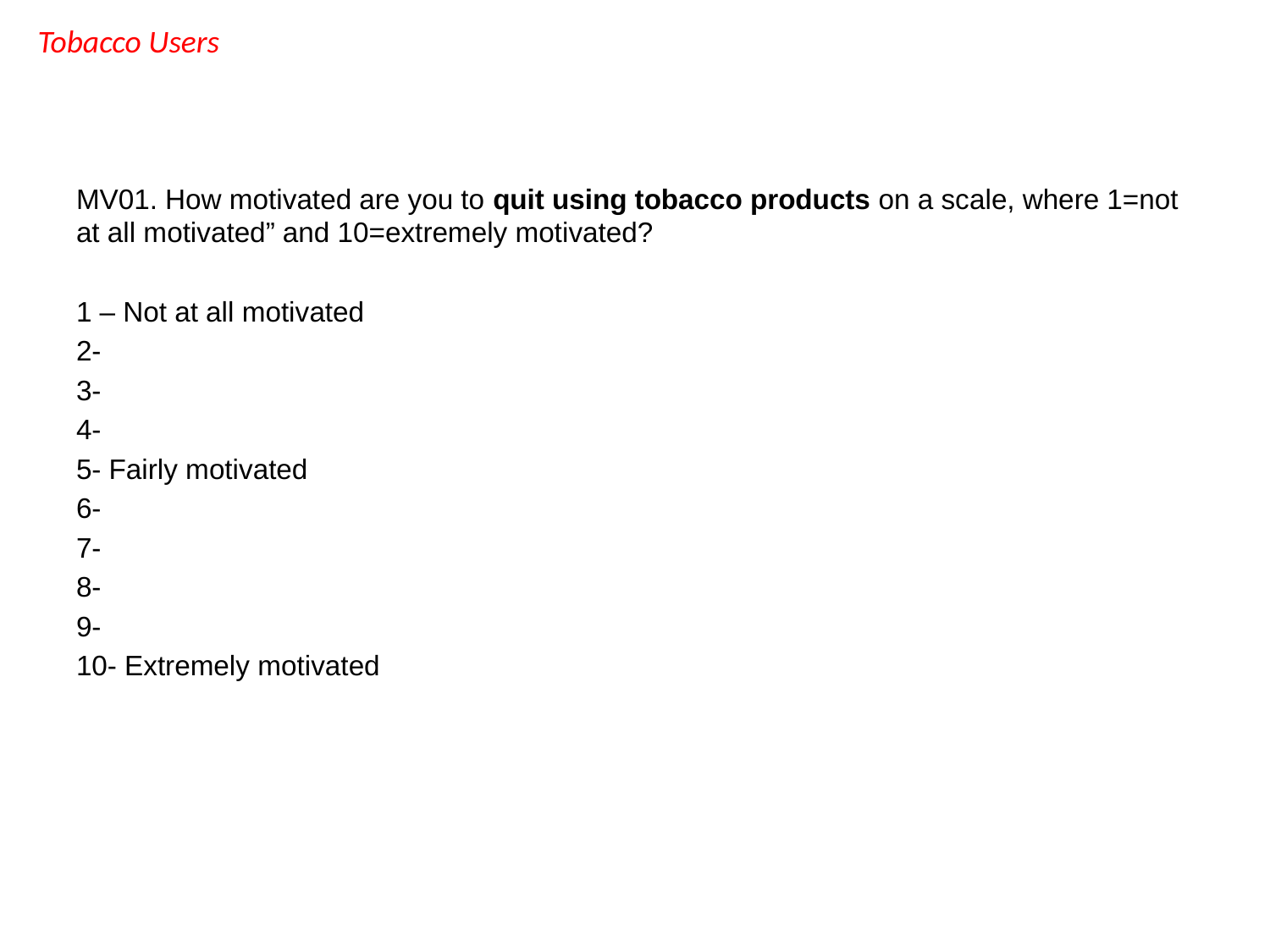

Tobacco Users
MV01. How motivated are you to quit using tobacco products on a scale, where 1=not at all motivated” and 10=extremely motivated?
1 – Not at all motivated
2-
3-
4-
5- Fairly motivated
6-
7-
8-
9-
10- Extremely motivated

## Slide 5
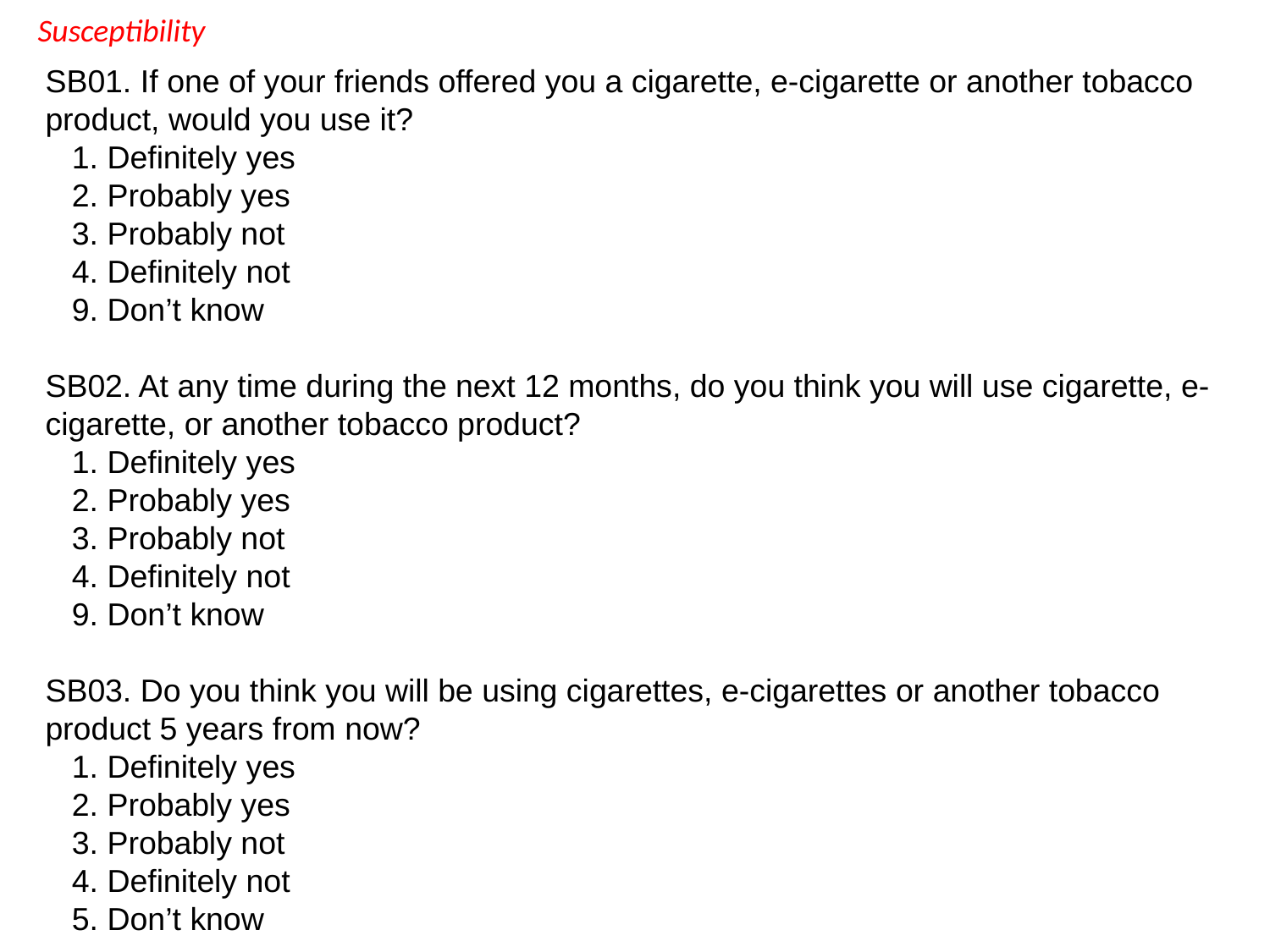

Susceptibility
SB01. If one of your friends offered you a cigarette, e-cigarette or another tobacco product, would you use it?
 1. Definitely yes
 2. Probably yes
 3. Probably not
 4. Definitely not
 9. Don’t know
SB02. At any time during the next 12 months, do you think you will use cigarette, e-cigarette, or another tobacco product?
 1. Definitely yes
 2. Probably yes
 3. Probably not
 4. Definitely not
 9. Don’t know
SB03. Do you think you will be using cigarettes, e-cigarettes or another tobacco product 5 years from now?
 1. Definitely yes
 2. Probably yes
 3. Probably not
 4. Definitely not
 5. Don’t know

## Slide 6
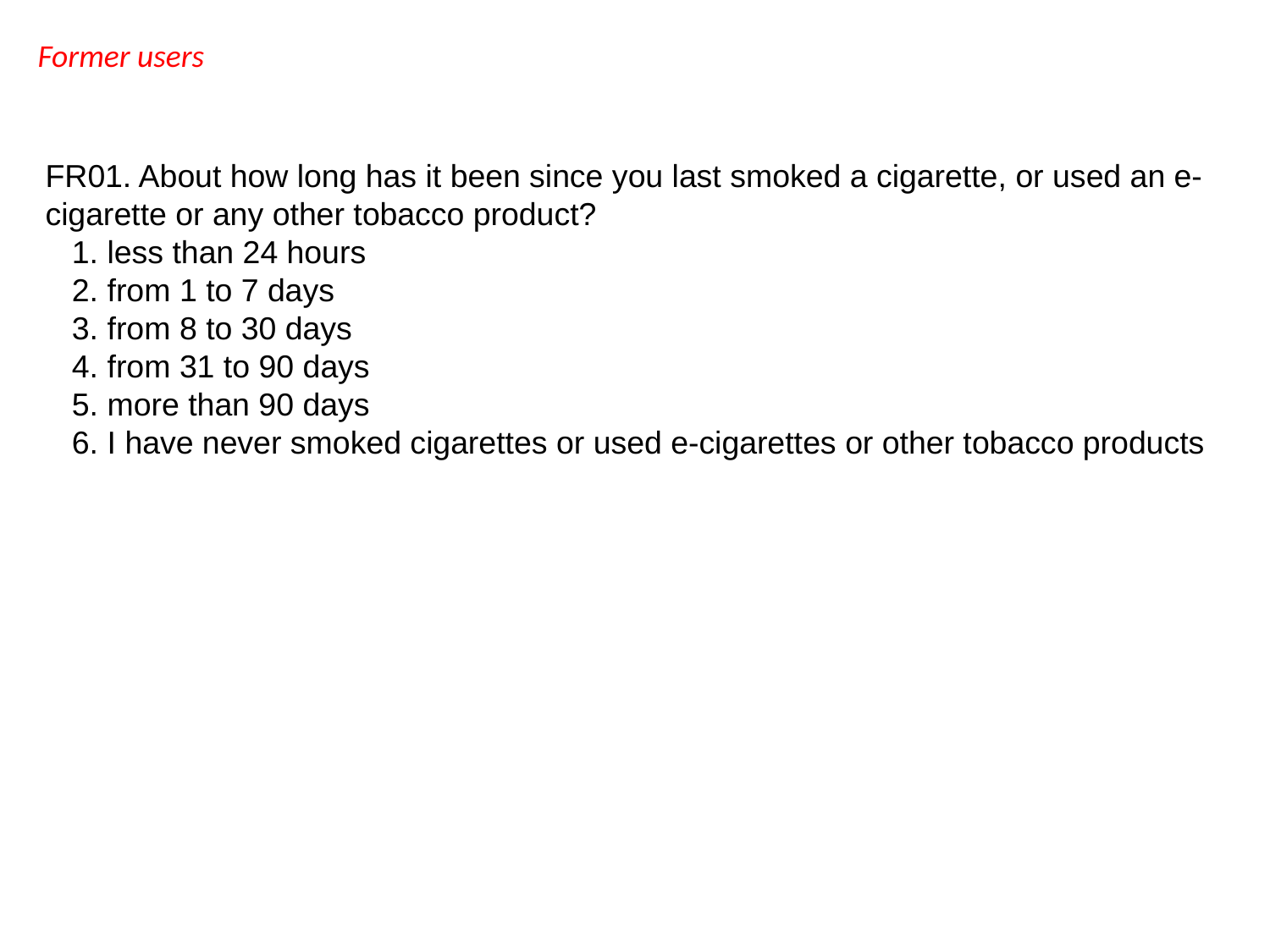

Former users
FR01. About how long has it been since you last smoked a cigarette, or used an e-cigarette or any other tobacco product?
 1. less than 24 hours
 2. from 1 to 7 days
 3. from 8 to 30 days
 4. from 31 to 90 days
 5. more than 90 days
 6. I have never smoked cigarettes or used e-cigarettes or other tobacco products

## Slide 7
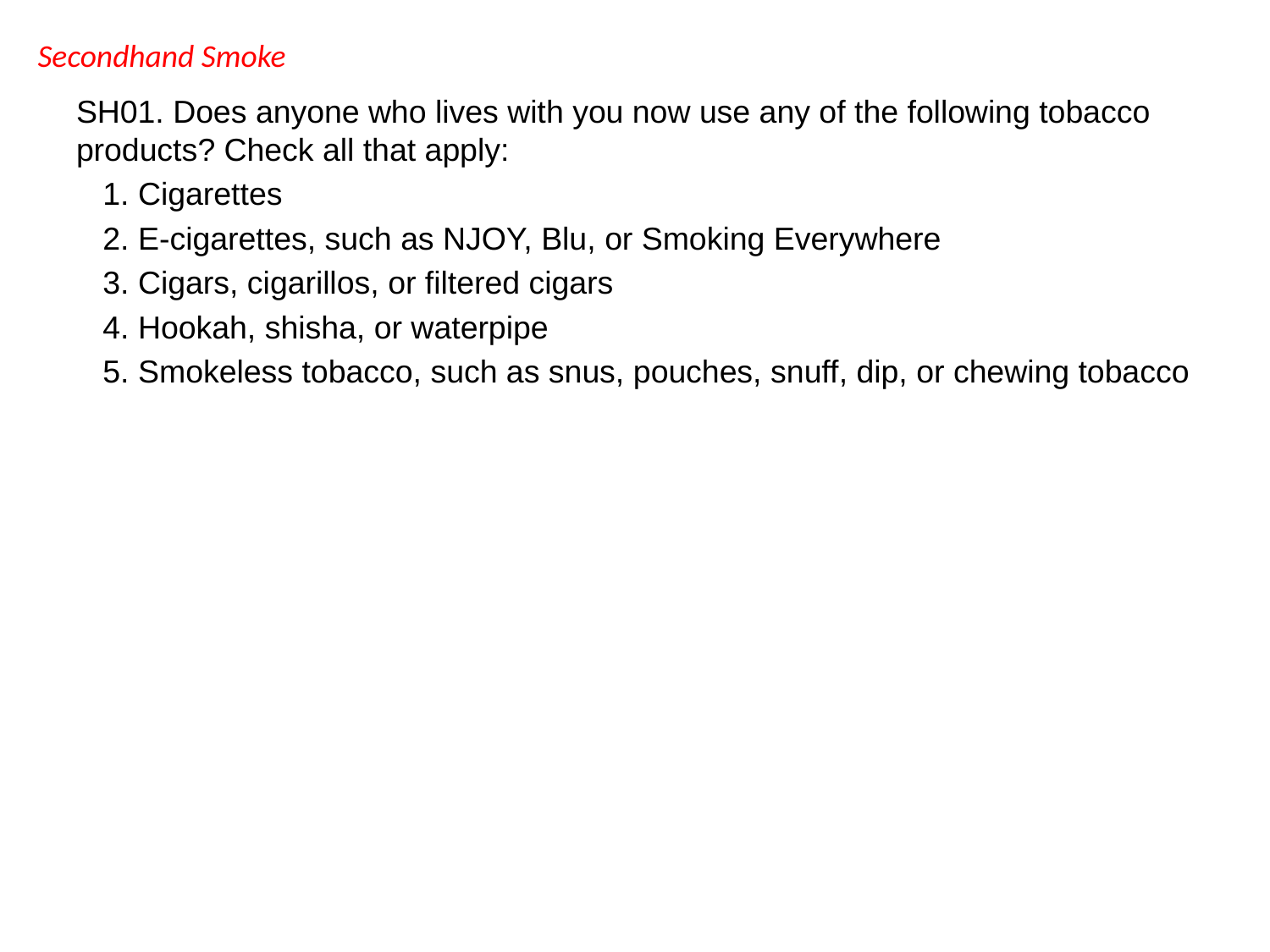

Secondhand Smoke
SH01. Does anyone who lives with you now use any of the following tobacco products? Check all that apply:
 1. Cigarettes
 2. E-cigarettes, such as NJOY, Blu, or Smoking Everywhere
 3. Cigars, cigarillos, or filtered cigars
 4. Hookah, shisha, or waterpipe
 5. Smokeless tobacco, such as snus, pouches, snuff, dip, or chewing tobacco

## Slide 8
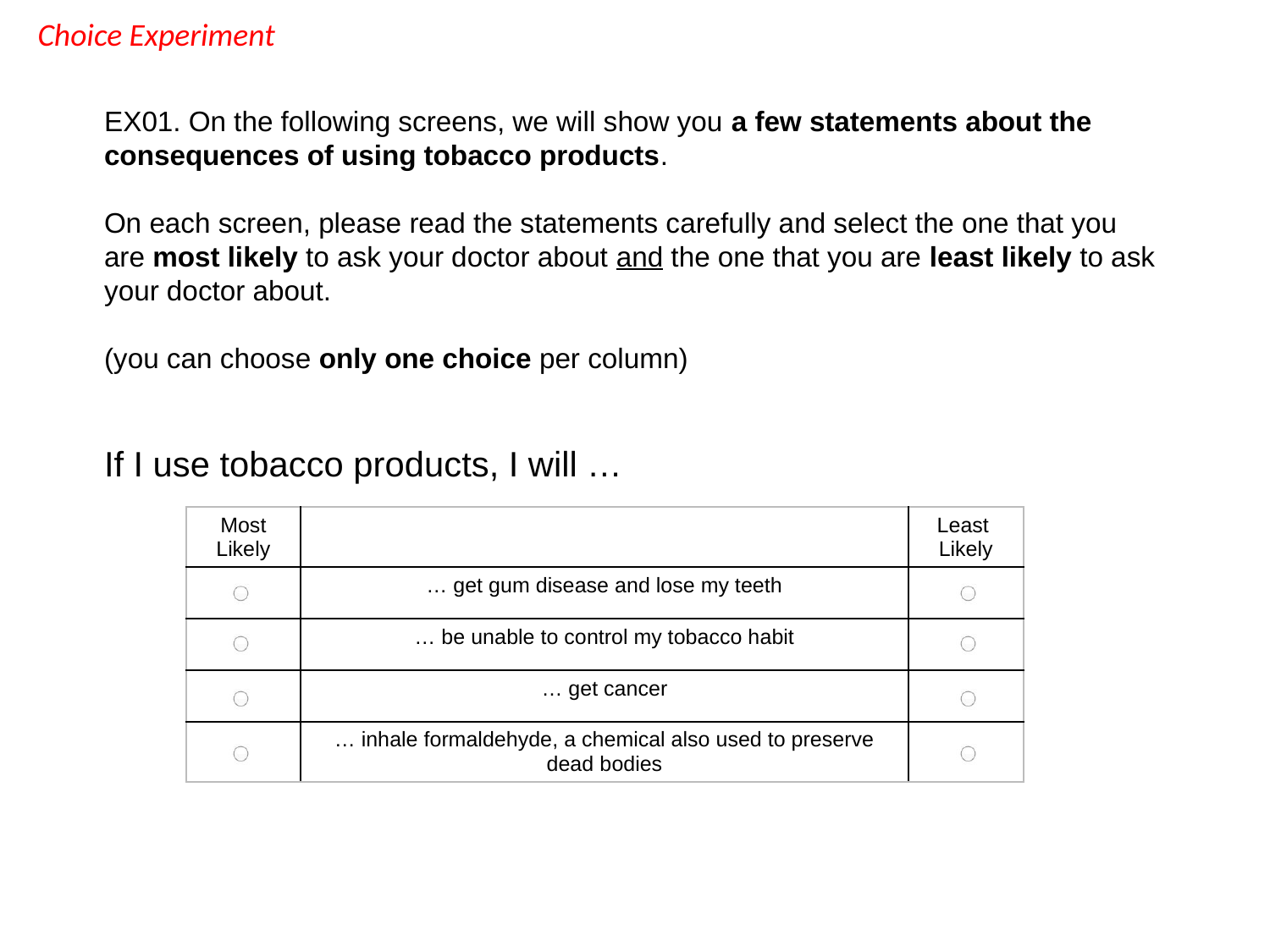

Choice Experiment
EX01. On the following screens, we will show you a few statements about the consequences of using tobacco products.
On each screen, please read the statements carefully and select the one that you are most likely to ask your doctor about and the one that you are least likely to ask your doctor about.
(you can choose only one choice per column)
If I use tobacco products, I will …
| Most Likely | | Least Likely |
| --- | --- | --- |
| | … get gum disease and lose my teeth | |
| | … be unable to control my tobacco habit | |
| | … get cancer | |
| | … inhale formaldehyde, a chemical also used to preserve dead bodies | |

## Slide 9
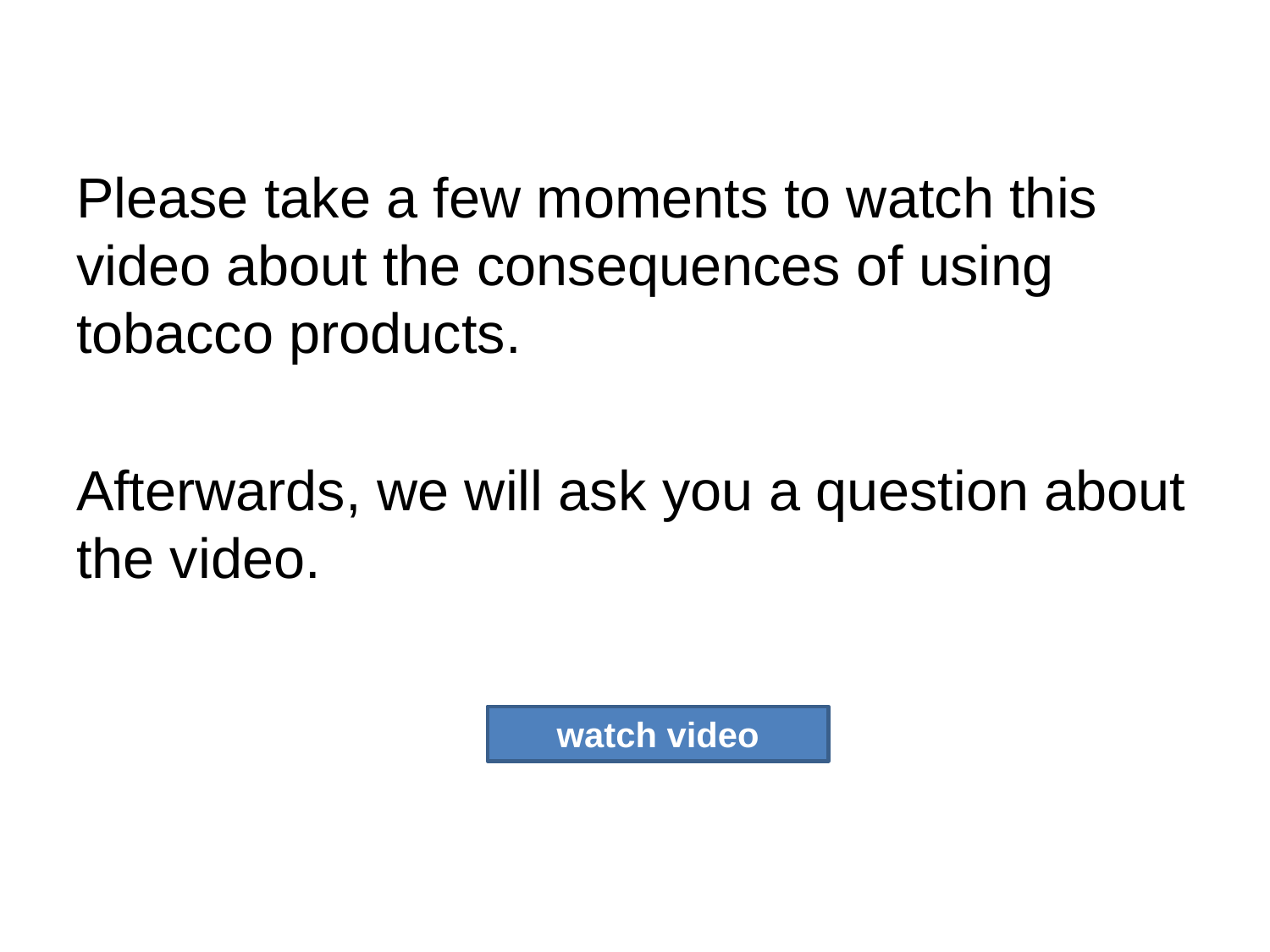

Please take a few moments to watch this video about the consequences of using tobacco products.
Afterwards, we will ask you a question about the video.
watch video

## Slide 10
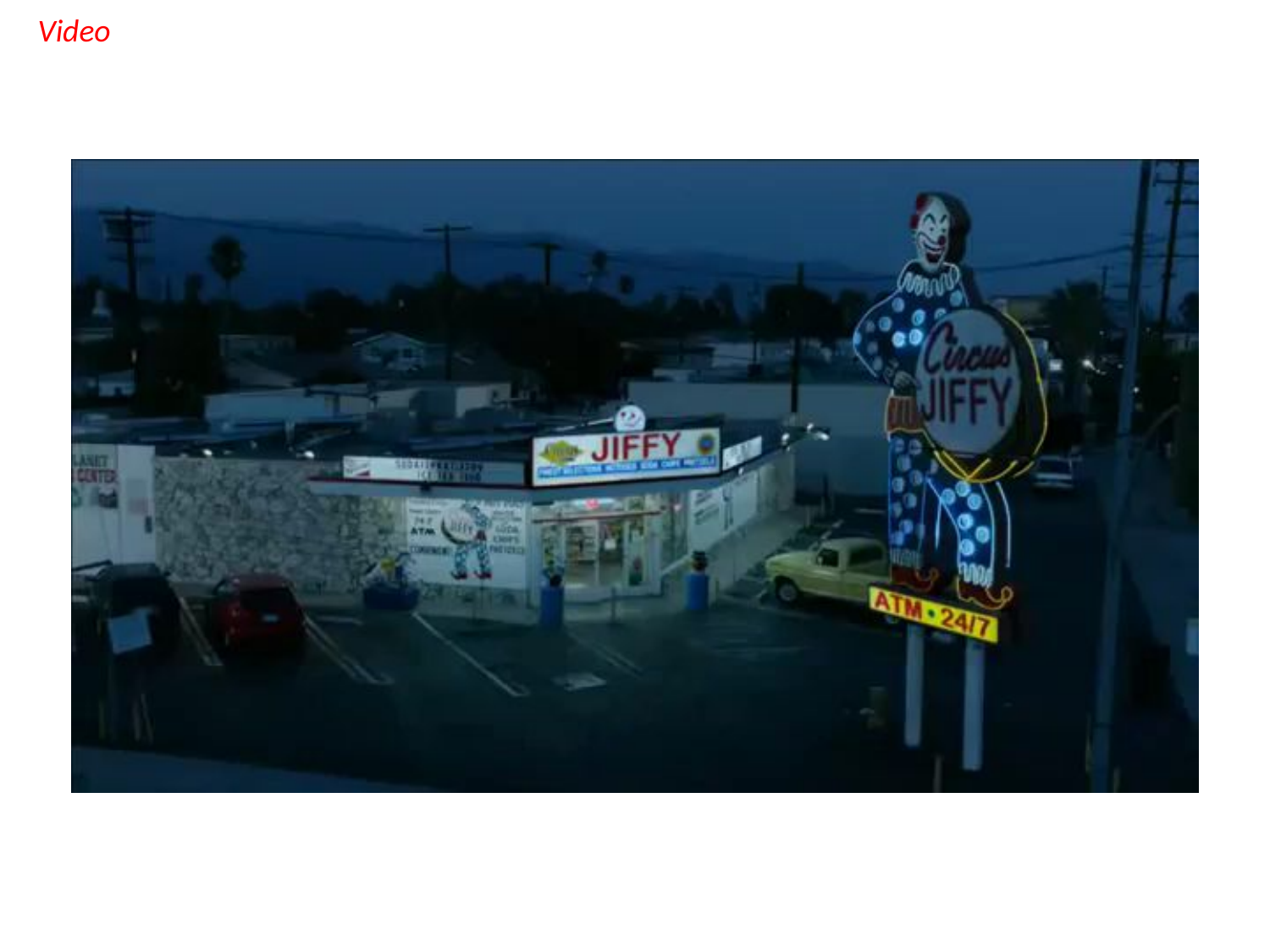

Video

## Slide 11
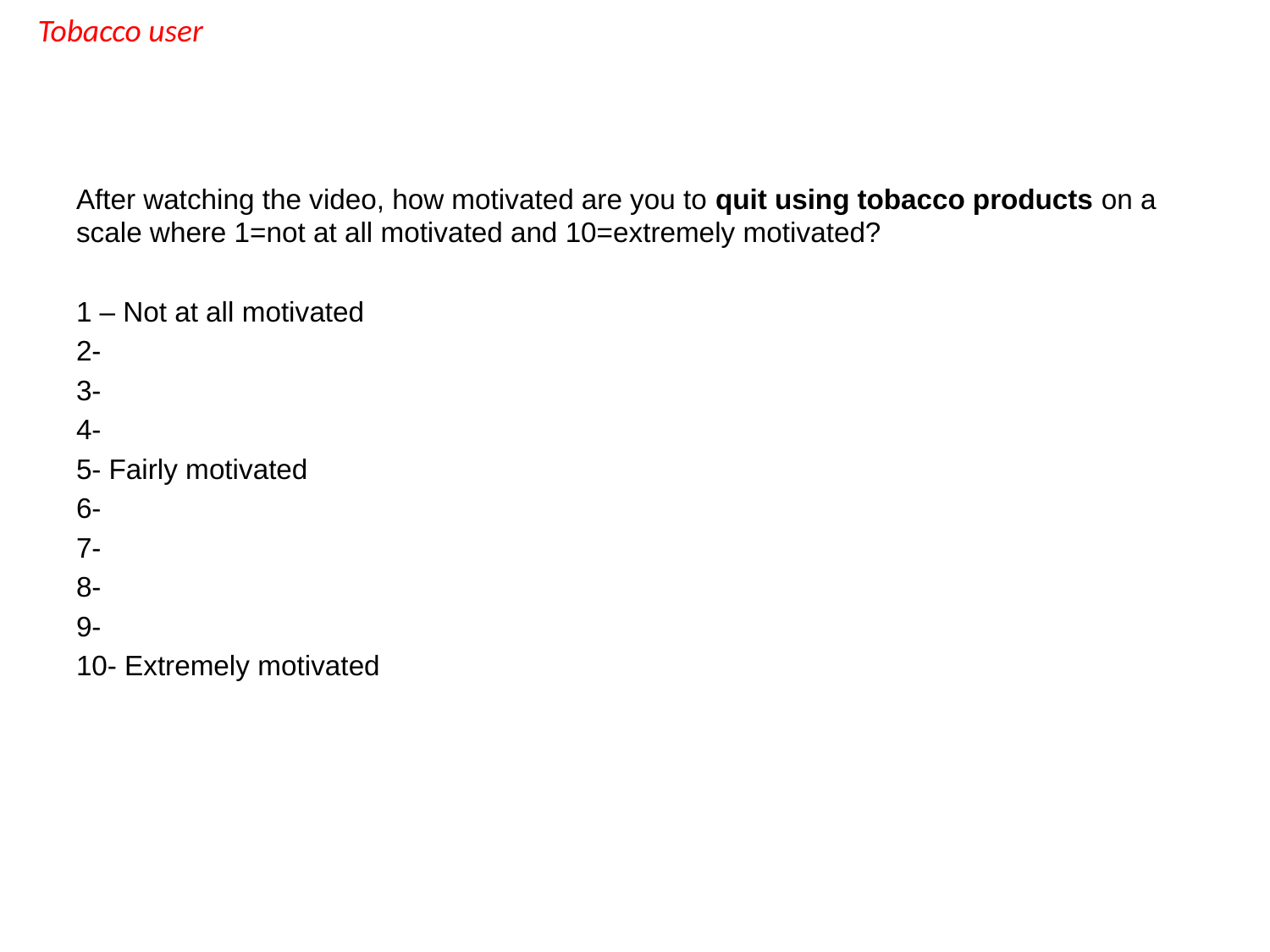

Tobacco user
After watching the video, how motivated are you to quit using tobacco products on a scale where 1=not at all motivated and 10=extremely motivated?
1 – Not at all motivated
2-
3-
4-
5- Fairly motivated
6-
7-
8-
9-
10- Extremely motivated

## Slide 12
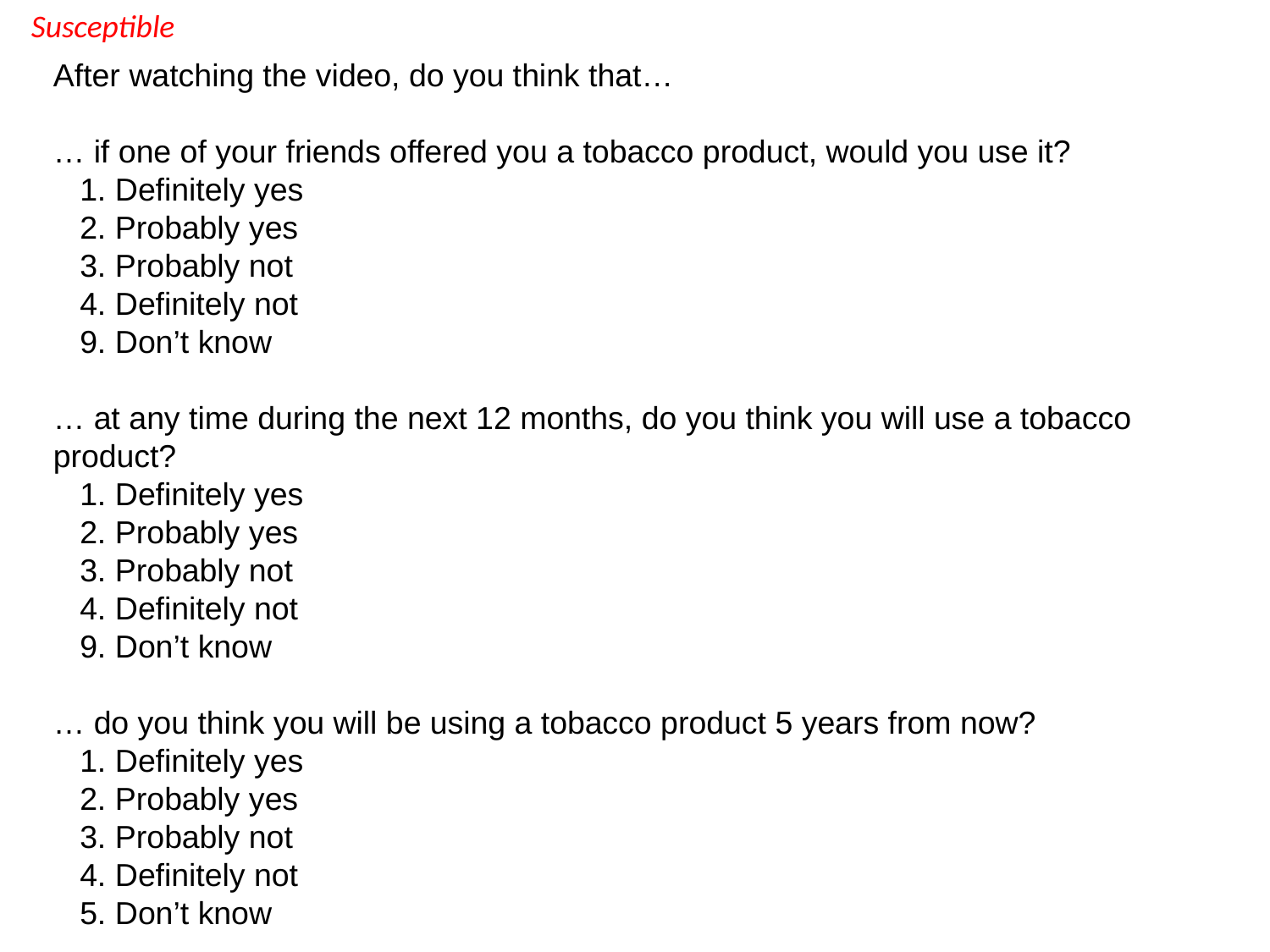

Susceptible
After watching the video, do you think that…
… if one of your friends offered you a tobacco product, would you use it?
 1. Definitely yes
 2. Probably yes
 3. Probably not
 4. Definitely not
 9. Don’t know
… at any time during the next 12 months, do you think you will use a tobacco product?
 1. Definitely yes
 2. Probably yes
 3. Probably not
 4. Definitely not
 9. Don’t know
… do you think you will be using a tobacco product 5 years from now?
 1. Definitely yes
 2. Probably yes
 3. Probably not
 4. Definitely not
 5. Don’t know

## Slide 13
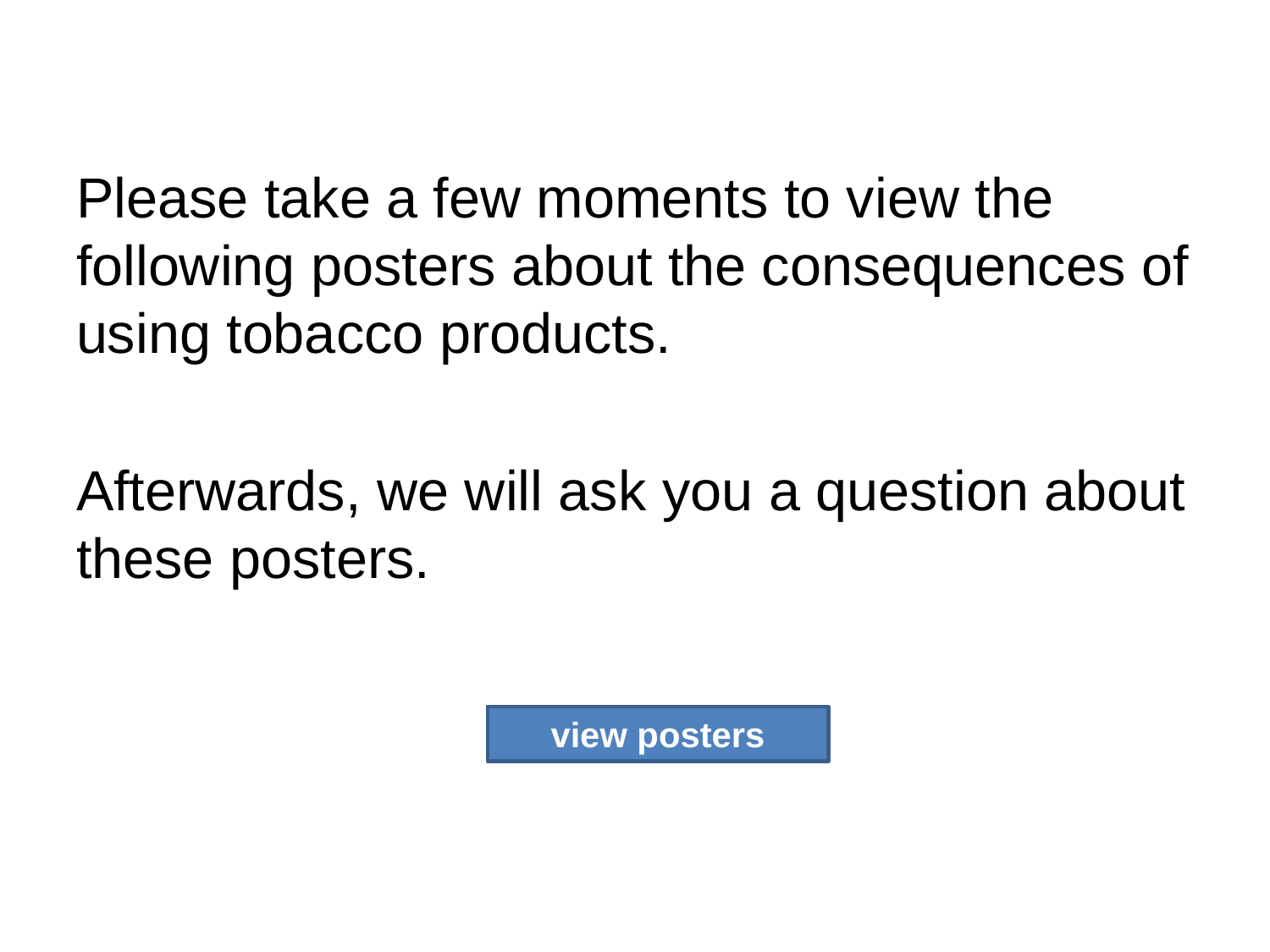

Please take a few moments to view the following posters about the consequences of using tobacco products.
Afterwards, we will ask you a question about these posters.
view posters

## Slide 14
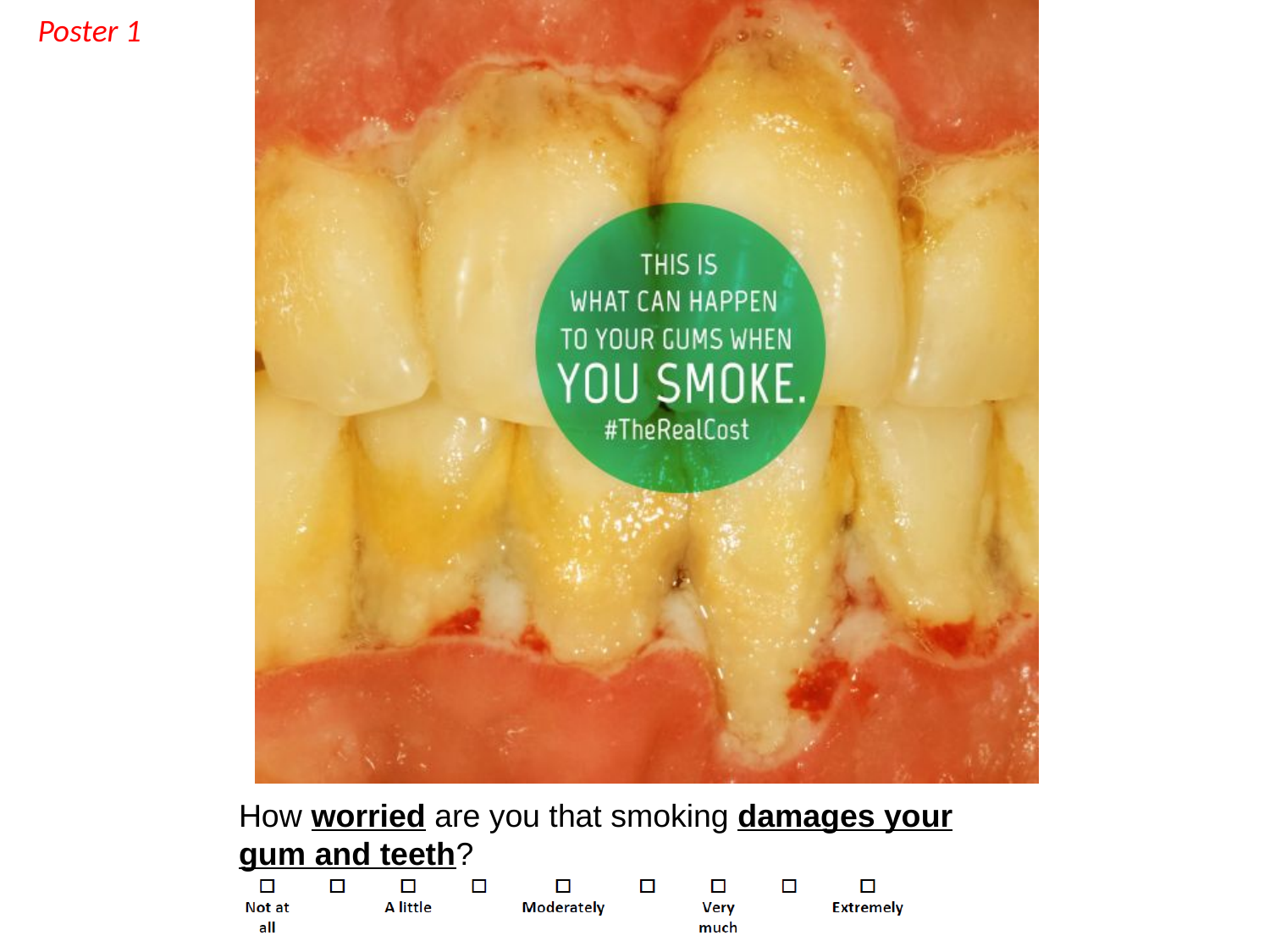

Poster 1
How worried are you that smoking damages your gum and teeth?

## Slide 15
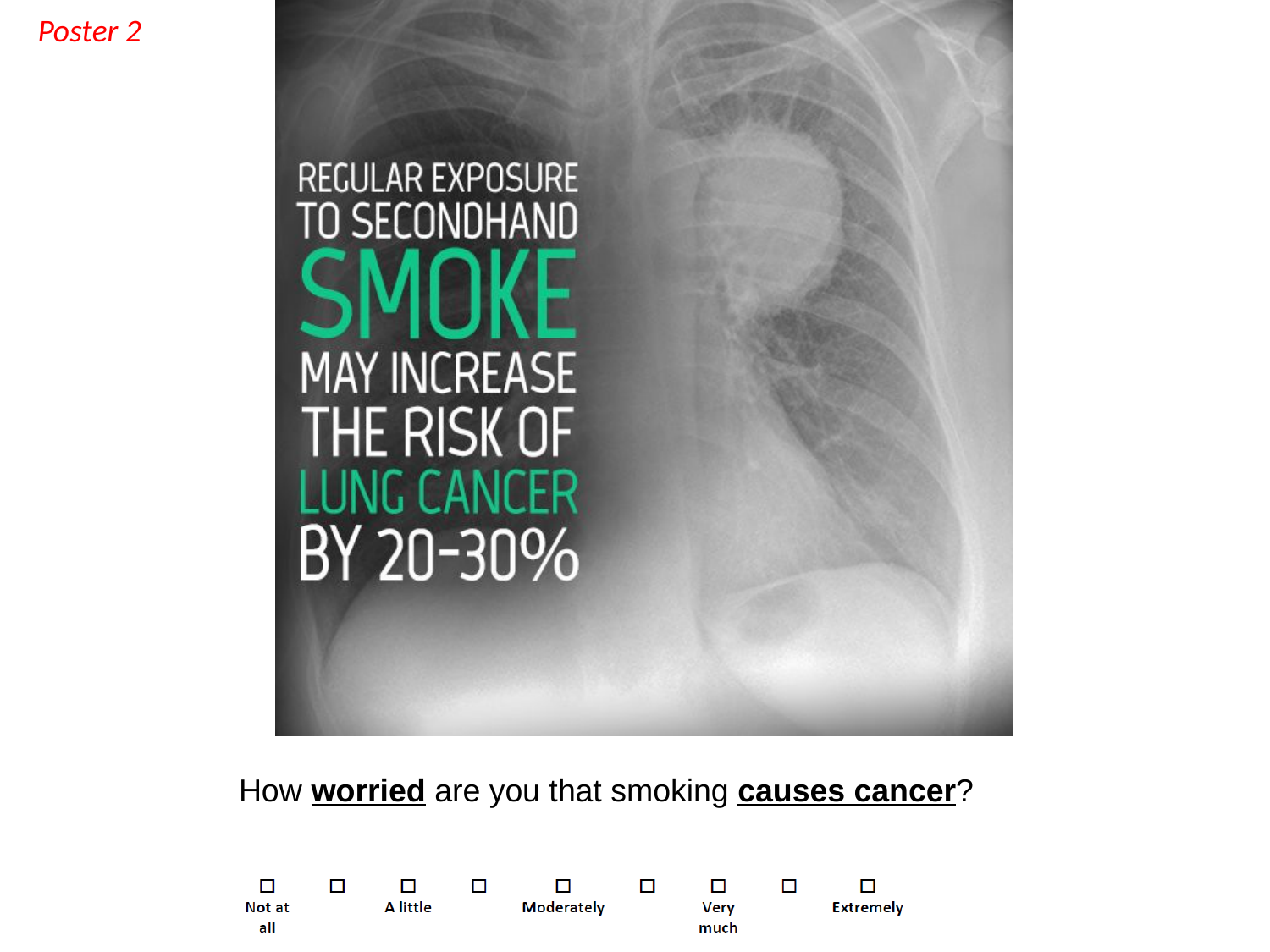

Poster 2
How worried are you that smoking causes cancer?

## Slide 16
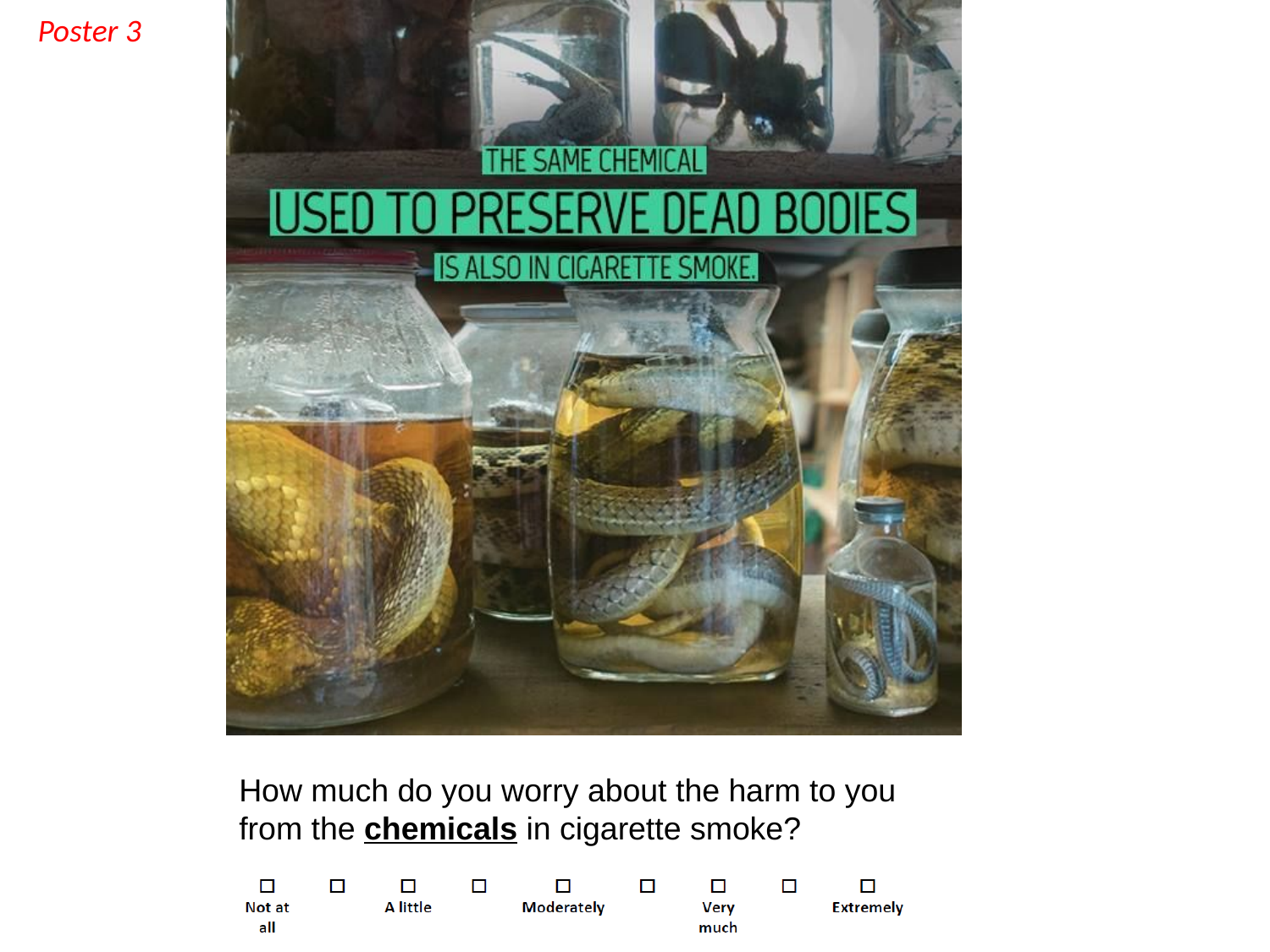

Poster 3
How much do you worry about the harm to you from the chemicals in cigarette smoke?

## Slide 17
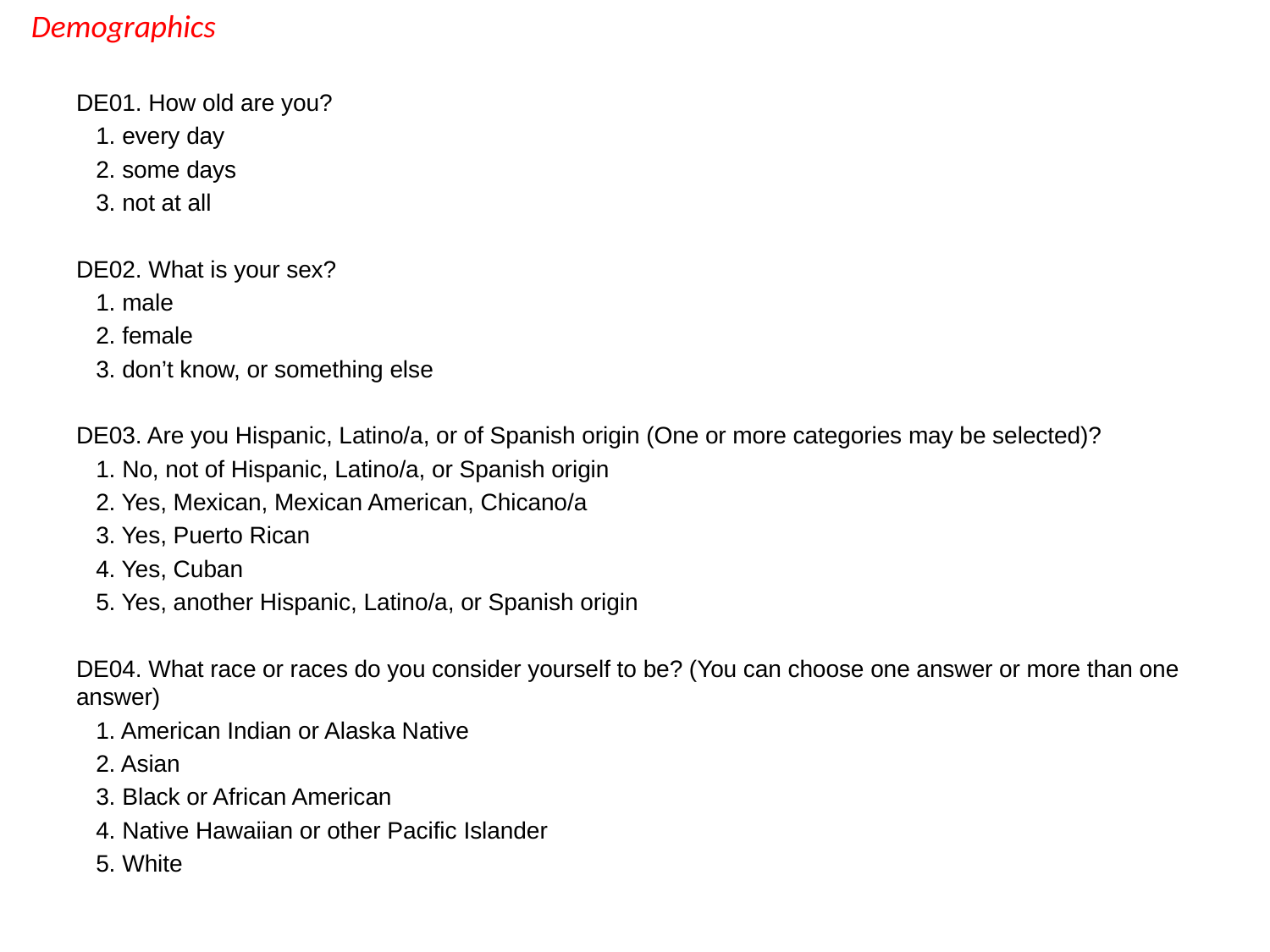

Demographics
DE01. How old are you?
 1. every day
 2. some days
 3. not at all
DE02. What is your sex?
 1. male
 2. female
 3. don’t know, or something else
DE03. Are you Hispanic, Latino/a, or of Spanish origin (One or more categories may be selected)?
 1. No, not of Hispanic, Latino/a, or Spanish origin
 2. Yes, Mexican, Mexican American, Chicano/a
 3. Yes, Puerto Rican
 4. Yes, Cuban
 5. Yes, another Hispanic, Latino/a, or Spanish origin
DE04. What race or races do you consider yourself to be? (You can choose one answer or more than one answer)
 1. American Indian or Alaska Native
 2. Asian
 3. Black or African American
 4. Native Hawaiian or other Pacific Islander
 5. White
